# Supplementary material for: Prevalence and epidemiological characteristics of COVID-19 after one year of pandemic in Jakarta and neighbouring areas, Indonesia: A single center study
Source: PLoS One. 2022 May 12;17(5):e0268241. doi: 10.1371/journal.pone.0268241 (PMC9098020; doi:10.1371/journal.pone.0268241)
Supplement: Attachment — Submitted filename: Response to Reviewers.docx [file pone.0268241.s002.docx]

**Rebuttal Letter**

PONE-D-21-28166R1

Prevalence and epidemiological characteristics of COVID-19 after one year of pandemic in Jakarta and neighbouring areas, Indonesia: A single center studyPLOS ONEDear

1. Some minor revision and clarification: The data in the text mention that: The province that contribute for more than 60% cases are Jakarta (21.3%), West Java (16.8%), Central Java (11.6%), East Java (9.4%), and East Kalimantan (3.7%). The figure 1 do not showed any data from East Kalimantan.

**RESPONSE:** Thank you for your comments. These data were cited from the National Committee for Corona Virus Diseases 2019 Handling and Economic Recovery reported on August 2021 (In discussion part: Line 155-161). Meanwhile, Figure 1 showed data for current study (Line 107-111). We cited the report from the National Committee to provide comparison (Reference No. 15). We have revised the text and added citation and reference accordingly.

2. Please add not only symptoms, but also radiological finding for pneumonia in the text and figure 3.

**RESPONSE:** Thank you for your suggestion. We have revised the text to include pneumonia based on chest x-ray results and figure 3 has been updated accordingly. Please see line 130-136 and Figure 3:

……..”Chest X-ray data were available from 845 out of 10130 positive cases, which were reported as: pneumonia 679/845 (80.4%), other abnormalities (including bronchitis, pleural effusion, lung abscess, and increased bronchovascular markings) 46/845 (5.4%), and normal 120/845 (14.2%). Most of SARS-CoV-2 cases were asymptomatic (74.0%). Among symptomatic cases of SARS-CoV-2, we found that the most common reported clinical symptoms (or combination of symptoms) (n=1,577) were cough (20.9%), a combination of both cough & fever (7.0%), and fever (6.8%) (Figure 3).”……..
